# The Value of the Albumin–Myosteatosis Gauge in Predicting the Postoperative Outcomes of Non-Metastatic Gastric Cancer

**DOI:** 10.3390/cancers18142333

**Published:** 2026-07-20

**Authors:** Huihui Zhang, Yunshi Huang, Jiacheng Jiang, Yuchen Wang, Xiangyang Xue, Xinxin Yang

**Affiliations:** 1 Department of Gastrointestinal Surgery, The First Affiliated Hospital of Wenzhou Medical University, Wenzhou Medical University, Wenzhou 325035, China; 2 Department of Microbiology and Immunology, Institute of Molecular Virology and Immunology, School of Basic Medical Sciences, Wenzhou Medical University, Wenzhou 325035, China; 3 Zhejiang Key Laboratory of Intelligent Cancer Biomarker Discovery and Translation, The First Affiliated Hospital of Wenzhou Medical University, Wenzhou Medical University, Wenzhou 325035, China; 4 Department of Clinical Nutrition, The First Affiliated Hospital of Wenzhou Medical University, Wenzhou Medical University, Wenzhou 325035, China

**Keywords:** myosteatosis, albumin, albumin–myosteatosis gauge, gastric cancer

## Abstract

**Background**: The potential synergistic prognostic value of myosteatosis and albumin levels in patients with gastric cancer (GC) remains to be elucidated. This study evaluates the value of the albumin–myosteatosis gauge (AMG) and Global Leadership Initiative on Malnutrition (GLIM) score in predicting postoperative complications and survival in patients who have undergone radical resection for GC. **Methods**: The present study enrolled patients diagnosed with stage I–III GC who underwent subtotal or total gastrectomy. Skeletal muscle area (SMA) and skeletal muscle density (SMD) were measured at the CT scan closest to the surgical procedure, which was performed for clinical diagnostic purposes. The AMG was defined as SMD × albumin. In this study, patients were divided into specific quartiles according to the AMG and GLIM. A series of analyses was conducted with the objective of identifying the risk factors associated with postoperative outcomes following GC surgery. The construction of a nomogram model for predicting prognosis was undertaken, and calibration curve analysis was utilised. **Results**: This study demonstrated that AMG is a more reliable predictor of OS than single indicators such as serum albumin and muscle density in patients with non-distant metastatic GC. Furthermore, the combination of AMG and GLIM scores has greater predictive value for GC outcomes and can be used as a novel prognostic scoring index in a GC patient prognostic model reflecting cachexia and malnutrition risk. **Conclusions**: The construction of a nomogram by our study team constitutes a simplified, reliable and efficient model for the prediction of the prognosis of GC, exhibiting considerable discriminative and calibration abilities.

## Simple Summary

Current reports indicate that 60–80% of patients with gastric cancer (GC) develop cachexia, which ultimately leads to elevated cancer-specific mortality and increased treatment-related toxicity. In the context of precachexia, a close correlation has been observed between systemic inflammation and muscle wasting. The present study set out with the objective of systematically evaluating the prognostic performance of the albumin–myosteatosis (AMG) as a simple and effective method of predicting overall survival (OS) among patients with GC, with a view to enhancing survival outcomes, given its clinical value.

## 1. Introduction

Gastric cancer (GC) is one of the most common and lethal malignancies worldwide [1,2]. At present, GC usually involves comprehensive treatment strategies, including surgery, chemotherapy and immunotherapy. These have significantly improved outcomes, although the prognosis remains poor. Numerous studies have currently focused on identifying clinical and treatment-related factors associated with the prognosis of patients with GC [3]. However, there is still no simple, effective method of accurately predicting the prognosis of patients with GC, which could help with treatment and prognosis.

Cachexia is a complex metabolic syndrome characterised by progressive weight loss, muscle wasting, systemic inflammation, altered energy metabolism and anorexia [2]. It leads to functional impairment and a poor prognosis [4]. It is commonly associated with chronic diseases, particularly cancer. Sarcopenia is highly prevalent among patients with upper gastrointestinal cancers, particularly in advanced malignant tumours [5,6]. Meanwhile, the definition of sarcopenia used by the European Working Group on Sarcopenia in Older People (EWGSOP) focuses on a decrease in muscle mass and quality. This is partly reflected by the deposition of intermuscular or intramyocellular fat, known as myosteatosis [7]. Current reports indicate that 60–80% of patients with cancer develop cachexia during disease progression [8,9], ultimately leading to elevated cancer-specific mortality and increased treatment-related toxicity. There has been extensive research into the role of sarcopenia (a marker of skeletal muscle atrophy and cachexia) in prognosis, which has paved the way for the exploration of other CT-derived muscle parameters with prognostic potential [10]. Early detection and intervention at the precachexia stage are crucial for achieving better clinical outcomes in such cases. A simple and effective method of accurately predicting the prognosis of patients with GC would have profound implications for enhancing survival outcomes, given the clinic value.

Systemic inflammation is a prognostic biomarker for outcomes in GC and a critical driver of muscle wasting pathogenesis [11,12,13]. It disrupts muscle homeostasis by impairing progenitor cell function and altering protein turnover, while also suppressing hepatic albumin synthesis, which is a key indicator of malnutrition and inflammatory status [14]. These parallel effects suggest an intertwined relationship between muscle quality and albumin metabolism [15], which is likely to be mediated through shared inflammatory pathways [16,17]. Regrettably, the potential synergistic prognostic value of muscle composition and albumin levels in patients with GC remains underexplored [18].

To address this, we employed the albumin–myosteatosis gauge (AMG), a novel composite metric calculated as follows: AMG = serum albumin (g/dL) × skeletal muscle density (SMD, in Hounsfield units). This study aimed to systematically evaluate the prognostic performance of the AMG against conventional biomarkers in predicting overall survival (OS) among patients with GC.

## 2. Methods

### 2.1. Patients Population

This retrospective study enrolled patients with GC who underwent subtotal or total gastrectomy at the Department of Gastrointestinal Surgery, First Affiliated Hospital of Wenzhou Medical University, China, between December 2014 and December 2019.

The following criteria apply: (1) patients who underwent a preoperative abdominal CT scan within 2 weeks and a serological examination within 1 week before surgery, and (2) patients willing to participate in the study and provide informed consent.

The following patients were excluded: (1) patients with a history of muscle diseases, haematological system diseases or kidney disease; (2) patients with severe preoperative infection; (3) patients with incomplete medical records; (4) patients undergoing palliative surgery or those with metastatic gastric cancer; and (5) patients who underwent neoadjuvant chemotherapy. The experimental flow chart is shown in Figure S1. According to the inclusion criteria, data relating to 1188 patients was collected. The study was approved by the First Affiliated Hospital of Wenzhou Medical University’s Institutional Review Board.

### 2.2. Patient Characteristics

Data on patient characteristics were obtained from a review of electronic medical records (EMRs) and included the following:

- Demographic information, such as gender, age, American Society of Anesthesiologists (ASA) grade, Charlson Comorbidity Index (CCI) and body mass index (BMI)
- Tumour characteristics, such as location, size, and histological grade
- Haematology tests, such as serum albumin levels
- Long-term overall survival (any causes of deaths), postoperative complications and readmission within one month after surgery (the postoperative outcomes aspect).

### 2.3. Measurement of Skeletal Muscle Index and Skeletal Muscle Radiodensity

Skeletal muscle area (SMA) and skeletal muscle density (SMD) were measured at the CT scan closest to the surgery, which was performed for clinical diagnostic purposes.

### 2.4. Albumin-Myosteatosis Gauge

The albumin–myosteatosis gauge (AMG) was calculated using the following formula: serum albumin (g/dL) × SMD (HU).

### 2.5. Defining Low and High Levels of AMG

According to the X-Tile software (3.6.1 version), AMG was categorised as low or high with cutoffs of 868.7 for females and 1377.6 for males.

### 2.6. Follow-Up

All patients were required to return for the necessary examinations within the first month after surgery. After that, they were followed up every three to six months for further examinations as needed for five years postoperatively. The follow-up programme consisted of physical examinations, laboratory tests and ultrasonography, CT scans and/or endoscopies.

Data on patient mortality, including the time and cause of death, was obtained primarily through medical records, telephone follow-up, and the local population database. The overall survival rate was determined as the proportion of patients who survived after surgery for GC. Postoperative complications were recorded within a month of surgery and classified using the Clavien–Dindo classification system. The last follow-up took place in December 2019.

### 2.7. Statistical Analysis

According to X-Tile software, AMG was categorised as low risk or high risk, with cutoffs of 868.7 and 1377.6 for females and males, respectively. The Kolmogorov–Smirnov test was used to determine whether the continuous data conformed to a normal distribution. Data that conformed to normal distribution were presented as mean and standard deviation, while data that did not conform to normal distribution were presented as median and interquartile range. Categorical variables were presented as quantity and percentage. The Student’s *t*-test was used to compare data conforming to a normal distribution. The Mann–Whitney U-test was used to compare non-normally distributed data, and the Pearson chi-squared test or Fisher’s exact test was used to compare categorical data.

The outcome of this study was overall survival, which was calculated from the date of surgery to the date of death or the last available follow-up date. Patients with OS periods longer than five years were censored. The Kaplan–Meier method was used to analyse overall survival and the log-rank test was performed to compare differences in survival between subgroups. Multivariate Cox proportional hazards regression analysis was performed to determine independent risk factors for long-term overall survival. Variables with *p* < 0.10 in univariate analysis were included in multivariate analysis and ultimately, variables with *p* < 0.05 were retained in the multivariate model. All tests were two-tailed, with statistical significance set at *p* < 0.05. All statistical analyses were performed using R version 4.1.0, and statistical significance was set at *p* < 0.05.

The patients were divided into training and validation cohorts at a ratio of 7:3, using the R function ‘create Data Partition’ to ensure the outcome events were distributed randomly between the two cohorts.

The training cohort was then utilised to select the variables required for constructing the model. The validation cohort was subsequently utilised to corroborate the findings derived from the training cohort.

Univariate and multivariate Cox regression analyses were performed on all individual variables. Variables with *p* values below 0.05 in both analyses were identified as independent risk factors. Ultimately, all independent risk factors were included and plotted as a nomogram.

## 3. Results

### 3.1. Baseline Characteristics

The study sample comprised 1188 patients with stage I–III GC. The median values of AMG differed considerably according to gender. Using X-tile software, we divided AMG into two subgroups (G0, low-AMG groups and G1, high-AMG groups), categorising patients as male or female. The AMG cutoffs were 1377.6 and 868.7 for female and male patients, respectively. There were 696 and 492 patients with GC in groups G0 and G1, respectively.

**Figure 1:**
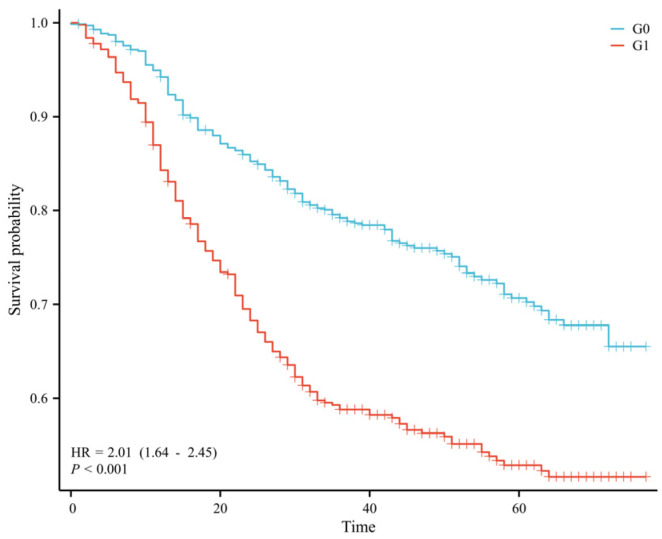
Kaplan–Meier survival curve. The 5-year overall survival for G0 (low-risk AMG groups) and G1 (high-risk AMG groups) are 24.6% and 20.7%, respectively (G0 vs. G1, *p* < 0.0001).

As shown in Table 1, the correlations between the high- and low-AMG groups and the following clinicopathological characteristics were determined: gender, age, BMI, NRS grade, ASA grade, tumour location, tumour size, histologic grade, complications, albumin level, SMI and SMD. Patients in the low-AMG group were older (years) (70.60 ± 8.19, *p* < 0.001), had a higher NRS-2002 grade (5–6 grades, 19.92%, all *p* < 0.001) and higher ASA grade (grades III and IV, 19.92%, *p* < 0.001), had a larger tumour size (≥5 cm, 38.41%, *p* < 0.001), experienced more complications (43.17%, *p* < 0.001), had lower mean serum albumin levels (g/dL) (35.20 ± 4.35, *p* < 0.001), lower mean SMI (cm^2^/m^2^) (131.68 ± 22.81, *p* < 0.001), and lower mean SMD (HU) (30.10 ± 6.58, *p* < 0.001) than those in the high-AMG group (Figure 1).

We compared SMD and albumin levels among the high- or low-AMG groups in males and females. There were significant differences in albumin level and SMD values according to the AMG group in both males and females (Figure S1, all *p* < 0.001).

### 3.2. The Kaplan–Meier (K-M) Survival Curve Categorised by AMG Group

K-M analysis revealed significant differences in 5-year overall survival (OS) between the high- and low-risk AMG groups (Figure 1, G0—24.6%; G1—20.7%, hazard ratio (HR) = 1.999 (1.638–2.438), *p* < 0.0001).

We further analysed the K-M survival curves separately in both males and females, and obtained consistent conclusions (Figure S2, all *p* < 0.001).

### 3.3. Univariable and Multivariable Analyses of Factors Associated with OS

In univariate analysis, the following were found to be significant: gender (*p* = 0.019), age (*p* < 0.001), BMI (*p* < 0.001), NRS score (*p* < 0.001), ASA grades (*p* < 0.001), CCI (*p* < 0.001), hypoalbuminaemia (*p* < 0.001), SMI (*p* < 0.001), tumour location (diffuse type vs. Antrum, *p* < 0.001), tumour size (*p* < 0.001), histologic grade (*p* < 0.001), TNM stage (all *p* < 0.001) and AMG (*p* < 0.001) differed significantly (Table 2). All *p* < 0.1 variables from the univariate analysis were included in the multivariate logistic regression analysis.

In conclusion, age (OR = 1.275, *p* < 0.001), tumour size (OR = 1.561, *p* < 0.001), TNM (II vs. I, OR = 1.766, *p* = 0.006; III vs. I, OR = 4.714, *p* < 0.001) and AMG1 (OR = 1.311, *p* = 0.034) were independently associated with GC.

In addition to the above results, we also evaluated the relationship between AMG and postoperative complications.

### 3.4. Category-Based Combination of AMG and GLIM

We divided AMG into four subgroups (G0: low-AMG and low-GLIM group, G1: high-AMG and low-GLIM group, G2: low-AMG and high-GLIM group, and G3: high-AMG and high-GLIM group) category-based combination of AMG and GLIM. There were 539, 318, 157 and 174 GC patients in groups G0, G1, G2 and G3, respectively.

As shown in Table 3, patients in the G3 group were older (≥37.4%, *p* < 0.001), had a higher NRS-2002 grade (≥5–6 grades, 19.92%, all *p* < 0.001) and higher ASA grade (grades III and IV, 19.92%, *p* < 0.001), had a larger tumour size (≥5 cm, 38.41%, *p* < 0.001), experienced more complications (43.17%, *p* < 0.001), had lower mean serum albumin levels (g/dL) (35.20 ± 4.35, *p* < 0.001), lower mean SMI (cm^2^/m^2^) (131.68 ± 22.81, *p* < 0.001), and lower mean SMD (HU) (30.10 ± 6.58, *p* < 0.001) than those in the high-AMG group.

### 3.5. Analysis of the Association Between the Category-Based Combination of AMG and GLIM and OS

After univariate analysis, variables with a *p*-value of less than 0.10 were included in the multivariate analysis. Multivariate analysis, adjusted for age, tumour size, tumour location, histologic grade and TNM level, identified AMG-GLIM as an independent prognostic factor for OS (G1 vs. G0, OR = 1.540 (1.184–2.003), *p* < 0.001; G2 vs. G0, OR = 1.506 (1.097–2.067), *p* = 0.011; G3 vs. G0, OR = 1.861 (1.387–2.497), *p* < 0.001, see Table 4).

### 3.6. Clinical Significance of Category-Based Combination of AMG and GLIM Index

**Figure 2:**
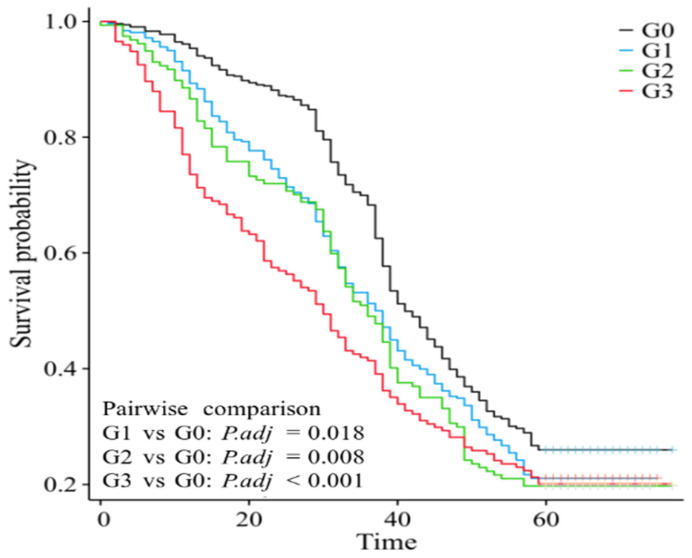
Kaplan–Meier survival curve. The 5-year overall survival for G0, G1, G2 and G3 were 26.0%, 21.1%, 20.1%, and 19.7%, respectively (G1 vs. G0, *p =* 0.018; G2 vs. G0, *p* < 0.01; G3 vs. G1, *p* < 0.001).

**Figure 3:**
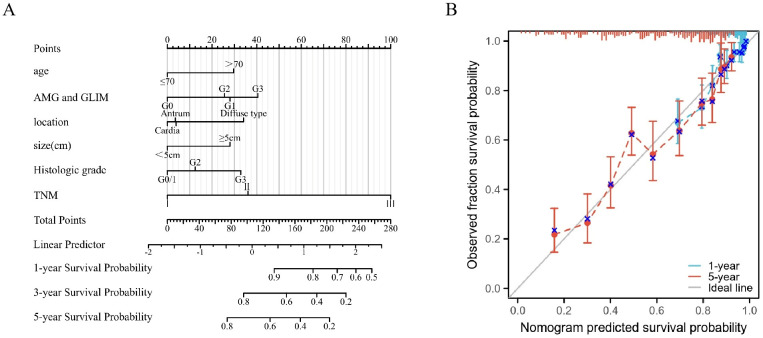
A constructed nomogram (**A**) and calibration curve (**B**) for the prognostic prediction of patients with GC.

As mentioned above, the patients were divided into four groups: G0, low-AMG and low-GLIM group; G1, high-AMG and low-GLIM group; G2, low-AMG and high-GLIM group; and G3, high-AMG and high-GLIM group. OS was significantly different among the four groups, as G0, G1, G2 and G3 were 26.0%, 21.1%, 20.1%, and 19.7% (respectively, *p* = 0.018, *p* < 0.01, and *p* < 0.001, Figure 2). We stratified the data into GLIM‑high and GLIM‑low subgroups, and within each subgroup, we further divided patients by AMG levels (high vs. low) for Kaplan–Meier survival analysis. As illustrated in Figure S3, there was a statistically significant difference in survival between the AMG‑high and AMG‑low groups (G0 vs. G1). Meanwhile, to compare the prognostic predictive value of the AMG index and the GLIM score for postoperative outcomes in gastric cancer, we performed Kaplan–Meier survival analyses across three groups: G0 (the entire cohort), G1 (the high-GLIM group), and G2 (the high-AMG group). The resulting survival curves are presented below; G0, G1, and G2 were 23.0%, 19.9%, and 20.7% (G1 vs. G0, *p* < 0.01, G2 vs. G0, *p* < 0.001, G2 vs. G1, *p* > 0.05, Figure S4). K-M survival analysis revealed no statistically significant difference between the high-AMG and high-GLIM subgroups. However, when the AMG index and GLIM score were integrated, the combined metric exhibited superior prognostic stratification for gastric cancer, as shown in Figure 3. This improvement suggests that AMG and GLIM may capture non-overlapping aspects of the disease, thereby complementing each other to enhance predictive performance. Univariable analysis according to categorical classification showed statistical significance (G1 vs. G0, G2 vs. G0 and G3 vs. G0, respectively, *p* < 0.0001), as did multivariable analysis (Table 4).

### 3.7. Nomogram Variable Screening

Multivariate regression analysis identified six variables (age, tumour size, tumour location, histology grade, TNM tumour stage and AMG with GLIM) as independent prognostic factors for GC (Table 4). According to the stepwise regression results, there was no collinearity between the screened variables in the training cohort. Based on these variables, we constructed a nomogram for GC. Figure 2 illustrates how to use the nomogram to predict the survival probability of an individual patient. The total score is determined based on the individual scores calculated using the nomogram.

The C-index value was 0.814 (95% CI, 0.769–0.860) in the training cohort and 0.837 (95% CI, 0.781–0.893) in the validation cohort. The time-dependent area under the curve (AUC) was greater than 0.7 for predicting OS in both cohorts (Figure 3A), indicating favourable discrimination by the nomogram. The nomogram’s calibration curves showed high consistency between the predicted and observed survival probabilities in both cohorts (Figure 3B). In summary, the GC nomogram demonstrated considerable discriminative and calibrating abilities.

## 4. Discussion

GC is etiologically intertwined with nutritional status, and mounting evidence supports the prognostic utility of inflammation- and nutrition-derived indices [19,20,21]. Nevertheless, existing prognostic instruments are often insufficiently streamlined or validated. Our findings indicate that the AMG index surpasses serum albumin and muscle density alone in predicting overall survival for non-metastatic GC. In addition, combining AMG with GLIM scores enhances outcome prediction and offers a novel metric to stratify patients at risk of cachexia and malnutrition. This proposition is further strengthened by recent observations linking AMG to neoadjuvant chemotherapy response in locally advanced disease [22], thereby reinforcing the clinical rationale of our postoperative prognostic framework.

Research emphasis is increasingly directed toward the tumour microenvironment rather than the tumour cells themselves, owing to its broader pathophysiological impact [23]. Notably, the AMG index has been associated with sensitivity to immune checkpoint inhibitor therapy, suggesting its biomarker potential [17,24]. Meanwhile, sarcopenia remains highly frequent in advanced malignancies, particularly in upper gastrointestinal cancers [25,26,27], underscoring the importance of precachexia detection and early intervention for improved patient survival [28]. Sarcopenia can be estimated using the SMI obtained from a cross-sectional CT image at the L level [29]. The EWGSOP currently defines sarcopenia in terms of muscle mass, strength and physical performance, all of which are partly reflected in the deposition of intermuscular or intramyocellular fat [7]. Myosteatosis is the infiltration of adipose tissue into skeletal muscle and is associated with muscle strength relative to size. As CT examinations and serum albumin examinations are routinely performed on most surgical patients with GC, the assessment of muscle quality could be widely studied [30]. Both myosteatosis and systemic inflammation are widely recognised as unfavourable prognostic factors in gastrointestinal cancers. Serum albumin levels reflect the degree of malnutrition and systemic inflammatory burden, both of which play a pivotal role in the onset and evolution of myosteatosis. While such associations have been explored in colorectal carcinoma [9,31,32], studies examining their combined effects in gastric cancer are notably limited. Cancer-associated inflammation is visible at the initial stages of neoplastic progression and can promote the development of early neoplasia into advanced cancer [21]. Serum albumin, which is produced in the liver and is abundant in the blood, is a well-known marker of systemic inflammation and nutritional status [33]. Indeed, studies suggest that inflammation has an adverse impact on the prognosis of patients with GC. Assessing inflammation counts may indicate systemic inflammation, which is a valuable prognostic marker in cancer [34,35]. A serum albumin level is closely related to an impaired nutritional status and has been reported to be associated with poor survival in patients with GC [36,37]. These findings suggest an intertwined relationship between muscle quality and albumin metabolism, likely mediated through shared inflammatory pathways [16,38].

As a novel composite parameter that couples serum albumin with skeletal muscle density, the AMG index was identified as an independent predictor of both early and late postoperative survival in multivariate regression models—a distinction not observed for albumin or muscle density alone. Based on these findings, we developed a prognostic model that incorporates age, tumour site, AMG index, GLIM category, TNM stage, and histologic grade. This model enables straightforward prediction of 1-, 3-, and 5-year overall survival in patients with GC, with calibration plots confirming its robust predictive performance.

The index model has distinct advantages in clinical practice. Firstly, albumin and abdominal CT scans are routine preoperative investigations for GC, and the AMG index can be easily acquired in clinical electronic medical records. Secondly, the model calculation method is straightforward, and the index exhibits evident accuracy and stability when predicting the survival of patients diagnosed with GC.

To further elucidate the interlink, prospective research, as well as pertinent fundamental research, could be conducted as follow-up investigations. We were unable to analyse the predictive value of changes in pre- and postoperative AMG levels over time; this is one of the limitations of this study.

## 5. Conclusions

The construction of a nomogram by the research team constitutes a simplified, reliable and efficient model for the prediction of the prognosis of GC, exhibiting considerable discriminative and calibration abilities.

The model integrates the following factors: age; tumour location; AMG index; GLIM index; TNM staging system; and histological grade. This approach enables the intuitive forecasting of OS in patients with GC, yielding satisfactory predictive outcomes as defined by calibration curve results. The index model has distinct advantages in clinical practice.

## Figures and Tables

**Table 1:** Baseline demographics and clinical characteristics of the gastric cancer study population, stratified by high and low AMG groups.

Variables	Total (*n* = 1188)	Low Risk AMG Group (*n* = 696)	High Risk AMG Group (*n* = 492)	*p*
Age	64.76 ± 10.77	60.64 ± 10.48	70.60 ± 8.19	<0.001
Gener				<0.001
Male	319 (26.85)	258 (37.07)	61 (12.40)	
Female	869 (73.15)	438 (62.93)	431 (87.60)	
BMI	22.54 ± 3.05	22.66 ± 2.95	22.37 ± 3.17	0.106
Haemoglobin	120.12 ± 23.76	127.15 ± 19.98	110.17 ± 25.11	<0.001
Albumin	38.02 ± 4.61	40.01 ± 3.66	35.20 ± 4.35	<0.001
SMD (HU)	34.95 ± 7.63	38.38 ± 6.36	30.10 ± 6.58	<0.001
SMA	133.45 ± 26.22	134.70 ± 28.34	131.68 ± 22.81	0.043
SMI	43.03 ± 8.05	43.83 ± 8.10	41.90 ± 7.84	<0.001
NRS-2002				<0.001
1–2	742 (62.46)	468 (67.24)	274 (55.69)	
3–4	327 (27.53)	185 (26.58)	142 (28.86)	
5–6	119 (10.02)	43 (6.18)	76 (15.45)	
ASA				<0.001
1–2	1045 (87.96)	651 (93.53)	394 (80.08)	
3–4	143 (12.04)	45 (6.47)	98 (19.92)	
CCI				0.002
0	689 (58.00)	428 (61.49)	261 (53.05)	
1	293 (24.66)	168 (24.14)	125 (25.41)	
≥2	206 (17.34)	100 (14.37)	106 (21.54)	
History of abdominal surgery				0.940
No	1030 (86.70)	603 (86.64)	427 (86.79)	
Yes	158 (13.30)	93 (13.36)	65 (13.21)	
Tumour location				0.012
Antrum	644 (54.21)	381 (54.74)	263 (53.46)	
Corpus	332 (27.95)	210 (30.17)	122 (24.80)	
Cardia	177 (14.90)	90 (12.93)	87 (17.68)	
Diffuse type	35 (2.95)	15 (2.16)	20 (4.07)	
Tumour Size				<0.001
<5 cm	832 (70.03)	529 (76.01)	303 (61.59)	
≥5 cm	356 (29.97)	167 (23.99)	189 (38.41)	
Histologic grade				0.004
G0/1	164 (13.80)	98 (14.08)	66 (13.41)	
G2	424 (35.69)	222 (31.90)	202 (41.06)	
G3	600 (50.51)	376 (54.02)	224 (45.53)	
TNM				<0.001
I	428 (36.03)	300 (43.10)	128 (26.02)	
II	249 (20.96)	142 (20.40)	107 (21.75)	
III	511 (43.01)	254 (36.49)	257 (52.24)	
Type of surgery				<0.001
Subtotal gastrectomy	736	469 (67.39)	267 (54.27)	
Total gastrectomy	452	227 (32.61)	225 (45.83)	
Complications				<0.001
No	524 (63.21)	316 (68.25)	208 (56.83)	
Yes	305 (36.79)	147 (31.75)	158 (43.17)	
Time	15.93 ± 9.69	14.90 ± 9.34	17.37 ± 9.99	<0.001
Cost	65,494.39 ± 29,564.02	61,305.19 ± 24,594.81	71,420.56 ± 34,593.16	<0.001

Abbreviations: BMI, body mass index; SMD, skeletal muscle density; HU, Hounsfield unit; SMI, skeletal muscle index; ASA, Anesthesiologists grade; CCI, Charlson Comorbidity Index.

**Table 2:** Univariable analysis of factors associated with overall survival.

	Total (*n*)	Univariate Analysis		Multivariate Analysis	
		HR (95% CI)	*p*	HR (95% CI)	*p*
Gender	1188				
Male	319	Reference		Reference	
Female	869	1.329 (1.047–1.686)	**0.019**	1.275 (0.986–1.649)	0.064
Age	1188				
≤70	1000	Reference		Reference	
>70	188	2.009 (1.596–2.530)	**<0.001**	1.574 (1.221–2.029)	**<0.001**
BMI	1188				
<20.5	308	Reference		Reference	
≥20.5	880	0.715 (0.578–0.885)	**0.002**	0.998 (0.767–1.298)	0.990
NRS-2002	1188				
1–2	742	Reference		Reference	
3–4	327	1.478 (1.181–1.850)	**<0.001**	1.048 (0.722–1.521)	0.805
5–6	119	2.674 (2.026–3.529)	**<0.001**	1.256 (0.803–1.965)	0.319
ASA	1188				
1–2	1045	Reference		Reference	
3–4	143	1.577 (1.214–2.048)	**<0.001**	1.189 (0.905–1.564)	0.214
CCI	1188				
0	689	Reference			
1	293	1.228 (0.975–1.548)	0.082		
≥2	206	1.221 (0.935–1.596)	0.143		
Abdominal surgery history	1188				
No	1030	Reference			
Yes	158	0.883 (0.653–1.195)	0.421		
Anaemic	1188				
No	748	Reference		Reference	
Yes	440	1.740 (1.428–2.120)	**<0.001**	0.868 (0.679–1.108)	0.256
Hypoalbuminaemia	1188				
No	899	Reference		Reference	
Yes	289	1.902 (1.546–2.341)	**<0.001**	1.090 (0.847–1.402)	0.502
GLIM	1188				
Low-group	857	Reference		Reference	
High-group	331	1.946 (1.588–2.384)	**<0.001**	1.239 (0.821–1.871)	0.307
AMG	1188				
Low-group	696	Reference		Reference	
High-group	492	2.006 (1.645–2.447)	**<0.001**	1.311 (1.020–1.686)	**0.034**
SMI	1188				
Low-group	835	Reference		Reference	
High-group	353	1.442 (1.173–1.773)	**<0.001**	0.981 (0.758–1.270)	0.886
Tumour location	1188				
Antrum	644	Reference		Reference	
Corpus	332	0.927 (0.731–1.175)	0.532	1.017 (0.798–1.295)	0.893
Cardia	177	1.028 (0.771–1.373)	0.849	0.921 (0.682–1.243)	0.590
Diffuse type	35	2.709 (1.745–4.204)	**<0.001**	1.571 (0.996–2.477)	0.052
size	1188				
<5 cm	832	Reference		Reference	
≥5 cm	356	2.818 (2.312–3.434)	**<0.001**	1.561 (1.253–1.946)	**<0.001**
Histologic grade	1188				
G3	600	Reference		Reference	
G2	424	0.654 (0.527–0.812)	**<0.001**	0.711 (0.570–0.888)	**0.003**
G0/1	164	0.295 (0.195–0.445)	**<0.001**	0.591 (0.382–0.914)	**0.018**
TNM	1188				
I	428	Reference		Reference	
II	249	2.514 (1.719–3.676)	**<0.001**	1.766 (1.181–2.641)	**0.006**
III	511	7.296 (5.340–9.969)	**<0.001**	4.714 (3.334–6.665)	**<0.001**

Statistical significance was set at *p* < 0.05, and such values are presented in bold.

**Table 3:** Baseline demographics and clinical characteristics of the gastric cancer study population, categorised according to AMG combination GLIM groups.

Variables	G0 (*n* = 539)	G1 (*n* = 318)	G2 (*n* = 157)	G3 (*n* = 174)	*p* Value
Gender					<0.001
male	358 (66.4%)	288 (90.6%)	80 (51.0%)	143 (82.2%)	
female	181 (33.6%)	30 (9.4%)	77 (49.0%)	31 (17.8%)	
Age					<0.001
<70	511 (94.8%)	238 (74.8%)	142 (90.4%)	109 (62.6%)	
≥70	28 (5.2%)	80 (25.2%)	15 (9.6%)	65 (37.4%)	
BMI					<0.001
≥20.5	72 (13.4%)	35 (11.0%)	98 (62.4%)	103 (59.2%)	
<20.5	467 (86.6%)	283 (89.0%)	59 (37.6%)	71 (40.8%)	
NRS					<0.001
1–2	468 (86.8%)	274 (86.2%)	0 (0.0%)	0 (0.0%)	
3–4	66 (12.2%)	33 (10.4%)	119 (75.8%)	109 (62.6%)	
5–6	5 (0.9%)	11 (3.5%)	38 (24.2%)	65 (37.4%)	
ASA					<0.001
1–2	505 (93.7%)	259 (81.4%)	146 (93.0%)	135 (77.6%)	
3–4	34 (6.3%)	59 (18.6%)	11 (7.0%)	39 (22.4%)	
CCI					0.040
0	332 (61.6%)	165 (51.9%)	96 (61.1%)	96 (55.2%)	
1	129 (23.9%)	85 (26.7%)	39 (24.8%)	40 (23.0%)	
≥2	78 (14.5%)	68 (21.4%)	22 (14.0%)	38 (21.8%)	
Abdominal surgery history					0.425
No	467 (86.6%)	282 (88.7%)	136 (86.6%)	145 (83.3%)	
Yes	72 (13.4%)	36 (11.3%)	21 (13.4%)	29 (16.7%)	
Haemoglobin	132(119.5, 142.5)	116 (95, 134)	123 (108, 135)	102.5 (87, 122.8)	<0.001
Anemic					<0.001
No	439 (81.4%)	152 (47.8%)	103 (65.6%)	54 (31.0%)	
Yes	100 (18.6%)	166 (52.2%)	54 (34.4%)	120 (69.0%)	
Hypoalbuminea					<0.001
No	439 (81.4%)	152 (47.8%)	103 (65.6%)	54 (31.0%)	
Yes	100 (18.6%)	166 (52.2%)	54 (34.4%)	120 (69.0%)	
GLIM					<0.001
Low	539 (100.0%)	318 (100.0%)	0 (0.0%)	0 (0.0%)	
High	0 (0.0%)	0 (0.0%)	157 (100.0%)	174 (100.0%)	
AMG					<0.001
High	539 (100.0%)	0 (0.0%)	157 (100.0%)	0 (0.0%)	
Low	0 (0.0%)	318 (100.0%)	0 (0.0%)	174 (100.0%)	
Location					0.003
Antrum	289 (53.6%)	167 (52.5%)	92 (58.6%)	96 (55.2%)	
Corpus	171 (31.7%)	77 (24.2%)	39 (24.8%)	45 (25.9%)	
Cardia	70 (13.0%)	65 (20.4%)	20 (12.7%)	22 (12.6%)	
Diffuse type	9 (1.7%)	9 (2.8%)	6 (3.8%)	11 (6.3%)	
Size					<0.001
<5 cm	419 (77.7%)	209 (65.7%)	110 (70.1%)	94 (54.0%)	
≥5 cm	120 (22.3%)	109 (34.3%)	47 (29.9%)	80 (46.0%)	
Histologic grade					0.001
G0/1	82 (15.2%)	49 (15.4%)	16 (10.2%)	17 (9.8%)	
G2	177 (32.8%)	137 (43.1%)	45 (28.7%)	65 (37.4%)	
G3	280 (51.9%)	132 (41.5%)	96 (61.1%)	92 (52.9%)	
TNM					<0.001
I	253 (46.9%)	104 (32.7%)	47 (29.9%)	24 (13.8%)	
II	112 (20.8%)	65 (20.4%)	30 (19.1%)	42 (24.1%)	
III	174 (32.3%)	149 (46.9%)	80 (51.0%)	108 (62.1%)	
Hospitalisation time	13 (10, 15.5)	14 (12, 19)	13 (10, 17)	15 (12, 21)	<0.001
Hospitalisation cost	5.6 × 10^4^ (4.9 × 10^4^, 6.5 × 10^4^)	6.3 × 10^4^ (5.2 × 10^4^, 7.6 × 10^4^)	5.8 × 10^4^ (4.8 × 10^4^, 7.1 × 10^4^)	6.8 × 10^4^ (5.7 × 10^4^, 8.1 × 10^4^)	<0.001

G0, low-AMG and low-GLIM group; G1, high-AMG and low-GLIM group; G2, low-AMG and high-GLIM group; G3, high-AMG and high-GLIM group.

**Table 4:** Multivariable analysis of factors associated with overall survival.

Characteristics	Total (*n*)	Univariate Analysis		Multivariate Analysis	
		Hazard Ratio (95% CI)	*p* Value	Hazard Ratio (95% CI)	*p* Value
Age	1188				
<70	1000	Reference		Reference	
≥70	188	2.009 (1.596–2.530)	**<0.001**	1.574 (1.230–2.013)	**<0.001**
AMG-GLIM	1188				
G0	539	Reference		Reference	
G1	318	2.028 (1.577–2.609)	**<0.001**	1.540 (1.184–2.003)	**0.001**
G2	157	2.028 (1.483–2.772)	**<0.001**	1.506 (1.097–2.067)	**0.011**
G3	174	3.260 (2.481–4.283)	**<0.001**	1.861 (1.387–2.497)	**<0.001**
Tumour location	1188				
Antrum	644	Reference		Reference	
Corpus	332	0.927 (0.731–1.175)	0.532	1.005 (0.791–1.277)	0.967
Cardia	177	1.028 (0.771–1.373)	0.849	0.946 (0.706–1.269)	0.713
Diffuse type	35	2.709 (1.745–4.204)	**<0.001**	1.662 (1.063–2.600)	**0.026**
Tumour size	1188				
<5 cm	832	Reference		Reference	
≥5 cm	356	2.818 (2.312–3.434)	**<0.001**	1.543 (1.249–1.905)	**<0.001**
Histologic grade	1188				
G3	600	Reference		Reference	
G2	424	0.654 (0.527–0.812)	**<0.001**	0.728 (0.584–0.908)	**0.005**
G0/1	164	0.295 (0.195–0.445)	**<0.001**	0.601 (0.389–0.928)	**0.021**
TNM	1188				
I	428	Reference		Reference	
II	249	2.514 (1.719–3.676)	**<0.001**	1.740 (1.168–2.592)	**0.006**
III	511	7.296 (5.340–9.969)	**<0.001**	4.658 (3.305–6.566)	**<0.001**

Statistical significance was set at *p* < 0.05, and such values are presented in bold.

## Data Availability

The original contributions presented in this study are included in the article/Supplementary Materials. Further inquiries can be directed to the corresponding author.

## Supplementary Materials

The following supporting information can be downloaded at: https://www.mdpi.com/article/10.3390/cancers18142333/s1, Figure S1: The flowchart of patient inclusion; Figure S2: Kaplan-Meier survival curve for males (A) and females (B); Figure S3: Kaplan-Meier survival curve for GLIM-low (A) and GLIM-high (B); Figure S4: Kaplan-Meier survival curve for total (G0), GLIM-high (G1), and AMG-high (G2).
