# Supplementary material for: The Value of the Albumin–Myosteatosis Gauge in Predicting the Postoperative Outcomes of Non-Metastatic Gastric Cancer
Source: Cancers (Basel). 2026 Jul 20;18(14):2333. doi: 10.3390/cancers18142333 (PMC13406968; doi:10.3390/cancers18142333)
Supplement: Supplementary file 1 [file cancers-18-02333-s001.zip › cancers-4256811-supplementary.pdf]

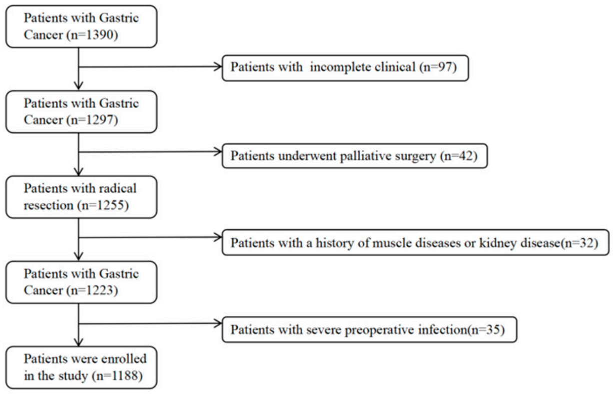

Figure S1: The flowchart of patient inclusion.

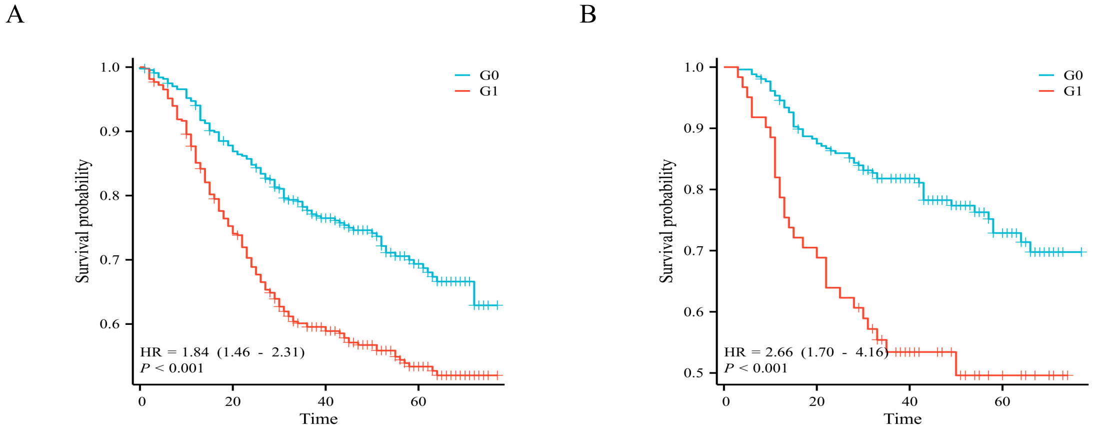

Figure S2: Kaplan-Meier survival curve for males (A) and females (B).

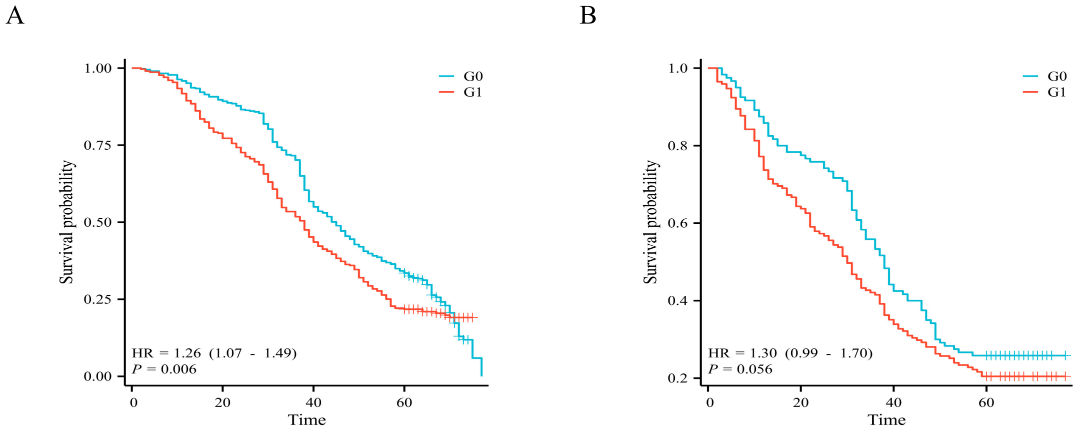

Figure S3: Kaplan-Meier survival curve for GLIM-low (A) and GLIM-high (B).

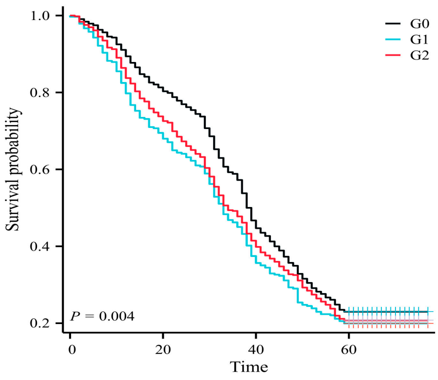

Figure S4: Kaplan-Meier survival curve for total (G0), GLIM-high (G1), and AMG-high (G2).
